# Discrepancies in Objective and Subjective Fine Motor Abilities in Octogenarians

**DOI:** 10.1093/geroni/igab046.3073

**Published:** 2021-12-17

**Authors:** Elizabeth Braungart Fauth, Andrew Hooyman, Sydney Schaefer, Anna Hall, Marie Ernsth-Bravell

**Affiliations:** 1 Utah State University, Logan, Utah, United States; 2 Arizona State University, Tempe, Arizona, United States; 3 Jönköping University, Jönköping, Jonkopings Lan, Sweden

## Abstract

Older individuals may have discrepancies between self-reported and performance-based abilities on activities of daily living (ADL). We examined objective and self-reported fine motor abilities (FMA). FMA are required for many ADLs, but are examined less frequently than gross-motor tasks in this population. We used two waves of the population-based OCTO-Twin study including mono-/dizygotic Swedish twins, aged 80+. One twin was randomly selected for analyses (baseline N=262; wave 2 N=198; Meanage =83.27; SDage=2.90; 66.4% female). Participants self-reported their ability to manipulate things with hands (cannot do, some problem, no problem) and completed a timed FMA assessment including five everyday tasks (e.g. inserting a key in a lock). Slow performance was coded as 1+ SD from the mean (=80+ seconds). At baseline, 65.8% of slow performers reported ‘no problems’ with hand manipulation. Over two waves (two years), a two-factor ANOVA (including slowness-by-perception interaction) supported a significant difference in total motor task performance between slow performers reporting ‘no problems’ and fast performers reporting ‘no problems’, for both rate of change (diff = -26 seconds, p<.0001) and wave 2 level (diff = 50 seconds, p < .0001). 82% of slow performers at wave 2 reported ‘no problems’, which is surprising given that they had become even slower over the past two years. Findings suggest that objective FMA measures are needed, as self-report is inaccurate and not prognostic. Future work will examine if discrepancies in performance/perceived FMA predict poorer outcomes, and/or if reporting ‘no problems’ despite slower performance is protective against cognitive adaptation to slowing.

